# Psychiatric adverse events reported after COVID-19 vaccination in the European Union (EU), the United States (US) and the United Kingdom (UK)

**DOI:** 10.1192/j.eurpsy.2022.230

**Published:** 2022-09-01

**Authors:** D. Macias Saint-Gerons, P. Correa Ghisays, R. Tabarés-Seisdedos

**Affiliations:** CIBERSAM/ISCIII, Incliva/universidad De Valencia, Valencia, Spain

**Keywords:** Covid-19, vaccine, adverse events

## Abstract

**Introduction:**

Several psychiatric adverse events can occur after vaccination. Passive surveillance reporting systems can support the identification of rare adverse events and contribute to hypothesis generation for potential causal associations.

**Objectives:**

To describe the psychiatric adverse reactions associated with various COVID-19 vaccines reported in the WHO database (VigiBase®)

**Methods:**

We for individual case reports (ICSRs) for “Psychiatric disorders” linked to COVID-19 vaccines authorized in the EU, the US and the UK. Reporting rates were calculated using the number of administered doses as a denominator. Disproportional reporting was investigated through frequentist and Bayesian approaches by the calculation the information component (IC) for adverse psychiatric adverse not included in the vaccine label.

**Results:**

63322 ICSRs including 76,163 psychiatric adverse events were identified, 21878 (34.6 %) were serious events. Mean age in the reports was 48.8 years old (SD: 17.8) and involved 44441 (70.2%) female and 17975 (28.4%) women; sex was not specified in 906 (1.4%) reports. Rate of reported psychiatric adverse events per million administered doses were 52.0, 110.3, 164.8 and 170.8 for Tozinameran/Cominarty (Pfizer-BioNTech), Elasomeran (Moderna), Vaxzevria (AstraZeneca) and Ad26.COV2-S (Janssen) vaccines respectively. UK recorded the highest rates. The most frequently reported events were insomnia (21.6%), confusional state (13.6%) and anxiety (13.5%). Disproportionality was found for: habit cough (IC:3.6), clinomania (IC: 2.2), exploding head syndrome (IC: 2.2) and autoscopy (IC: 2.1).

**Conclusions:**

Rates of reported psychiatric adverse events are very low. Doctors and patients should be aware of these potential adverse reactions. Continuing monitoring of emerging potential safety signals is advised.

**Disclosure:**

No significant relationships.

